# Husbandry Practices, Productive Performances, and Egg Quality Traits of SassoT44 and Koekoek Chicken Breeds in Urban and Rural Production Systems of Jama District, South Wollo Zone, Ethiopia

**DOI:** 10.1155/vmi/3025355

**Published:** 2025-03-12

**Authors:** Mesfin Fasil, Aleme Asresie, Yilkal Tadele

**Affiliations:** ^1^South Wollo Zone Agriculture and Fishery Development Office, Dessie, Ethiopia; ^2^Department of Animal Sciences, Wollo University, Dessie, Ethiopia; ^3^Department of Animal Sciences, Arba Minch University, Arba Minch, Ethiopia

**Keywords:** chicken, egg, Jama, performances, quality

## Abstract

Chicken production is an integral part of livestock farming in Ethiopia. Despite the local chicken ecotypes, the country introduces different exotic chicken breeds. This study aimed to assess husbandry practices, egg productive performances, and egg quality of SassoT44 and Koekoek chicken breeds in urban and rural production systems of Jama district. Purposive and random sampling techniques were employed to select 188 smallholder farmers for an interview. Data were collected on household characteristics, chicken ownership, husbandry practices, egg production performances, and egg quality traits. The proportion of males (59.3%) involved in chicken rearing is higher than that of females (40.7%). The results of the study indicated that majority of respondents (167) owned less number of SassoT44 and Koekoek chickens (5–10). Most of the respondents provided house and additional feed supplement to their chicken. The major feed sources were scavenging with supplements. The mean egg production performances of SassoT44 and Koekoek chicken breeds were 239.3 and 193 eggs/hen/year, respectively. Significantly higher (*p* < 0.001) numbers of eggs per hen/year (252.6 for SassoT44 and 198 for Koekoek) were recorded in the urban production system than in the rural production system (226 for SassoT44 and 187.5 for Koekoek). Egg weight was influenced by both chicken breed and production system. Higher (*p* < 0.001) weight of egg (56.7 g) was recorded for SassoT44 than that of Koekoek (51.1 g). SassoT44 was superior for a mean value of egg weight, width, albumen weight, yolk height, and yolk color (YC) than Koekoek. Most of the egg quality parameters have positive correlations. Sasso performs better in egg production and most egg quality traits in the study area.

## 1. Introduction

Poultry represents the largest segment of livestock globally, primarily comprising chickens, ducks, and turkeys [1]. Rural chickens play a crucial role in cultural practices, poverty reduction, and household food and nutritional security in many developing countries [2]. Ethiopia's total chicken population is estimated to be approximately 57 million, comprising 88.19% indigenous breeds, 6.45% hybrids, and 5.36% exotic breeds [3]. In Ethiopia, rural family poultry production systems offer significant opportunities for enhancing food security and nutrition, generating additional income, and empowering women [4, 5]. National poultry meat production of 7750 tons per year and total annual egg production of approximately 1.83 billion eggs, equivalent to around 73,357 tons, were reported [6].

The productivity of indigenous scavenging chickens in Ethiopia faces several constraints, including low egg laying capacity, high mortality rates, and lengthy reproductive cycles marked by slow growth, delayed sexual maturity, and extended broodiness. Moreover, the overall low output of these indigenous birds is partly attributed to inadequate management practices within traditional household poultry production systems [7]. Efforts have begun to incorporate various exotic poultry breeds into smallholder farming systems to enhance the performance of indigenous chicken breeds. However, the absence of documented data on husbandry practices and chicken performance poses challenges in evaluating the significance and contributions of these breeds [8].

SassoT44 and Koekoek (KK) have been introduced to smallholder farmers in Jama district. Nevertheless, management practices, egg productive performances, and egg quality of SassoT44 and KK breeds managed in urban and rural areas are not well studied and documented in the district. The management practices, egg production abilities, and egg quality of SassoT44 and KK chickens in both urban and rural areas remain undocumented. Addressing this information gap is crucial for identifying the challenges faced in chicken production and for implementing effective interventions. Therefore, this study was initiated with the objective to assess management practices, egg productive performances, and egg quality of SassoT44 and KK chicken breeds in rural and urban production systems of Jama district, Ethiopia.

## 2. Materials and Methods

### 2.1. Description of the Study Area

Jama District is one of the districts in South Wollo Zone, Amhara National Regional State, Ethiopia. It is located roughly from 10°39′59.99″N and 39°09′60.00″E latitudinal and longitudinal location. The district has 22 rural and two urban kebeles' administration. The total population and households are estimated above 149,925 and 35,691, respectively, out of which 15,836 were urban and 134,089 were rural inhabitants. It has an altitude range of 1428–2752 m above sea level (m.a.s.l), and it has two agroecologies, where 23% is lowland and 77% is midland. The average annual temperature ranges from 15 to 21.8°C. Its mean annual rainfall is about 1130 mm. The total population and households are estimated above 149,925 and 35,691, respectively, out of which 15,836 were urban and 134,089 were rural inhabitants. Chicken rearing is practiced in both urban and rural households in the area [9].

Several key factors were considered to select Jama district as a study site. There is no previous research regarding husbandry practices, productivity, and egg quality of exotic chicken in the district which highlights the need for this study. Similarly, higher number of exotic chicken breeds exists in the district which needs investigation. The willingness of local farmers and government offices to collaborate on the research initiatives was also considered.

### 2.2. Sampling Techniques and Sample Size

This study consisted two main parts: a survey and an analysis of egg quality traits. The survey was designed to collect data on management practices and egg production performance of KK chicken breeds in the district. The egg quality analysis aimed to evaluate the quality of eggs produced by these two breeds in the study area. Five kebeles were selected for the study; two from urban areas and three from rural areas, based on chicken production potential, breed availability, and road accessibility. Within each selected kebele, households owning at least five chickens of either SassoT44 or KK breeds were identified and listed. Respondents were then randomly selected from the list. The sample size was determined using Yemane's [10] formula, ensuring a 95% confidence level and a 5% margin of error. For the survey, a list of households raising the SassoT44 and KK breeds was obtained from the agriculture and fishery resource office of the district. Of the 354 households in the Jama district that adopted these breeds, 188 village households were selected for interviews, including 108 from rural and 80 from urban kebeles. *n*=*N*/1+*N*(*e*)^2^ where ‘‘*n*” is the sample size, ‘‘*N*” is the total population/total household, and ‘‘*e*” is the level of precision. *n*=(354/1+354 (0.05))2=∼188.

### 2.3. Data Sources and Collection Methods

A comprehensive and standardized questionnaire was developed for the household survey, covering a wide range of chicken-related activities in both urban and rural environments. A cross-sectional survey was conducted to gather information on household characteristics including gender and education level as well as the number and types of chickens owned, experiences in chicken holding and care, chicken housing and facilities, sources of feed and feeding systems, and production performance.

### 2.4. Analysis of Egg Quality Traits

A total of 120 eggs were collected to assess egg quality parameters of SassoT44 (60) and KK (60) chickens. The eggs for each breed were collected from both urban (30) and rural (30) production systems. Egg samples were collected from rural and urban households and then transported in an icebox with ice bags to the Debre Zeit Agricultural Research Center (DZARC) laboratory for the analysis of egg quality traits. Eggs were analyzed for internal and external egg quality parameters. The egg weight (EW) was individually weighed using a digital weighing balance. Egg width and egg length were measured by using digital vernier caliper meter. Then, eggs were broken onto a glass-covered table; albumen and yolk were separated and weighed each. Albumen and yolk heights were measured at its widest part using height meter (dial compressor gauge). Yolk diameter was measured horizontally using a digital caliper meter. Yolk color (YC) was determined by comparing it with the Roche Yolk Color (RYC) Fan, which contains 15 scales with a standard colorimetric system. The cleaned egg shell weight (SW) was weighed using a digital balance. The egg shell thickness was measured using the digital caliper meter determined by taking the average thickness of the large end, the center, and narrow ends. Furthermore, the other egg yolk index was calculated as Yolk height (mm)/Yolk diameter (mm).

### 2.5. Statistical Analysis

The collected data were analyzed using the Statistical Package for Social Sciences (SPSS, Version 25). Descriptive statistics were employed. Analysis of variance (ANOVA) was employed for analyzing the egg production performance and egg quality parameters using the general linear model (GLM) of SPSS. Means were separated using Tukey's test. Significant differences were declared at 5% significance level. The model used the following: *Yijk*=*µ*+*αi*+*βj*+(*αβ*)*ij*+*ℇijk*, where *Yijk* is the response variable, *µ* is the overall mean, *αi* is the effects of *i*^th^ chicken production systems (where *i* = 2, rural and urban), *βj* is the effects of *j*^th^ chicken breeds (2 = SassoT44 and KK), (*aβ*)*ij* is the interaction effect, and *ϵijk* is the random error.

## 3. Results

### 3.1. Household Characteristics

The household characteristics of interviewed urban and rural chicken owners are presented in Table 1. Out of the total respondents, 43.8% and 56.3% in urban and 59.3% and 40.7% in rural areas were male and female, respectively. The majority (64.9%) of the respondents were married.

### 3.2. SassoT44 and KK Chicken Holding and Keeping Experiences

SassoT44 and KK chicken breeds and keeping experiences of respondents are shown in Table 2. Only 7.5% and 18.5% of households had chicken keeping experiences of > 5 years in the urban and rural systems, respectively. The results of the study indicated that majority of respondents (167) owned less number of chickens (5–10) of SassoT44 and KK breeds.

### 3.3. Chicken Housing and Facilities

The findings on chicken houses and facilities are presented in Table 3. Majority of respondents, 76.3% in urban and 50% in rural areas, provide night shelter only to their chicken. Only few respondents, 7.5% in urban and 2.8% in rural, construct separate poultry houses.

### 3.4. Feed Sources and Feeding System

The results obtained on poultry feeds and feeding system used in the study areas are shown in Table 4. A higher proportion (79.25%) of respondents practices scavenging feeds with supplements. Similarly, a higher proportion (84.04%) of respondents provides feed supplements three times a day (morning, afternoon, and evening).

### 3.5. Egg Production Performances of SassoT44 and KK Chicken Breeds

Age at first lay, mature body weight, and egg production performances of SassoT44 and KK chicken breeds under urban and rural chicken production systems are shown in Table 5. There was a significant difference (*p* < 0.05) between two breed types and production systems in egg production performances. SassoT44 chickens surpassing KK chicken breeds in egg production might be due to a combination of genetic traits, their ability to adapt to the environment, and their performance on low-nutritional quality feeds, alongside traditional management practices.

### 3.6. External Egg Quality of SassoT44 and KK Chicken Breeds

The results on external egg quality observed in the current study are presented in Table 6. The present findings revealed that the EW and egg width of SassoT44 were significantly higher (*p* < 0.05) than that of KK chicken breed types. Similarly, EW, egg width, and egg shell thickness were higher (*p* < 0.05) in urban than in rural production systems.

### 3.7. Internal Egg Quality of SassoT44 and KK Chicken Breeds

The results of the laboratory analysis for internal egg quality traits are presented in Table 7. Albumen weight, yolk height, and yolk ratio were significantly different (*p* < 0.05) for Sasso and KK chicken breeds. Similarly, albumen and yolk weights (YWs) vary (*p* < 0.05) across production systems.

### 3.8. Correlation Coefficient of Egg Quality Parameters

The correlation coefficients among various egg quality parameters are shown in Table 8. The majority of these parameters exhibit positive correlations. Notably, EW shows strong positive correlations with all parameters, with the exception of albumen height (AH), albumen ratio (AR), and YC.

## 4. Discussion

The result of this study for the proportion of sex agreed with that of Ebsa, Harpal and Negia [11], Matawork et al. [12], and Habtie [13] who reported a higher proportion of male respondents participation in chicken production. Nega et al. [14] reported a higher proportion (88.2%) of married respondents in Assosa town, Ethiopia. Regarding educational background, Ebsa et al. [11] reported that 57% of the respondents did not attend any form of education in Dugda woreda, Ethiopia.

In the present study, the majority of respondents owned between five and 10 chickens, primarily of the SassoT44 and KK breeds. This observation corresponds with the findings of Ayalew et al. [15], who noted that producers in the northwestern regions of Ethiopia commonly raise commercial breeds such as SassoT44 and KK. Suntebo [16] found that 26.5%–51.3% of households provide nighttime shelter for their chickens in selected districts of the Gamo zone, Southern Ethiopia. In contrast, studies by Mohammed [17] and Aman et al. [18] reported that 50% and 43.8% of respondents, respectively, construct chicken houses in the Jigjiga zone and southern Ethiopia. This variation may be attributed to a lack of knowledge and awareness, insufficient materials, and inadequate attention to the significance of proper poultry housing.

In Ethiopia, village chicken production is predominantly characterized by a scavenging feeding system, which includes minimal supplementation [17]. Maize and wheat are the primary types of supplementary feeds for chickens. This finding aligns with the report,which identifies maize and wheat as common feed supplements, by Gonta et al. [19]. The popularity of these grains can be attributed to their local production by households, making them readily available for purchase. Over half of the respondents feed their chickens three times daily. Additionally, Gonta et al. [19] revealed that 29% of chicken owners also supplied supplementary feed to their chickens three times a day.

SassoT44 produced higher number of eggs (252.6) than KK (198) per year. Contrary to this, Aman et al. [18] reported an average egg production of 229.14 ± 52.49 eggs per hen per year for the Sasso breed within the village production system in southern Ethiopia. This could be attributed to different management practices in the study areas. Tadesse et al. [20] noted EW of 60.27 g for Bovans Brown (BB), 58.75 g for ISA Brown (IB), and 48.84 g for Potchefstroom KK (PK) chickens. Fanu et al. [21] also reported the average mean egg length of 58.38 mm, 56.46 mm, and 55.26 mm in Termaber, Ankober, and Kewet, respectively, for SassoT44 breed. The average egg shape index of this study falls into the normal egg category, which is 72–76. The shell thickness in the present study was comparable with that of Tadesse et al. [22], who reported 0.31 mm in East Shewa, Ethiopia. On the other hand, Kumar et al. [23] reported a higher value of shell thickness for Rhode Island Red (0.41 ± 0.04 mm) and Bovans Whites (0.39 ± 0.03 mm) in Mekelle, Ethiopia.

Nebiyu [24] noted AH of 7.1 ± 0.08 mm for BB breed in Addis Ababa. Taddesse et al. [22] also reported 18.11 ± 0.91 mm yolk height for BB breed under the village production system in East Shewa, Ethiopia. The present finding in yolk height is higher than 15.1 ± 1.3 mm as reported by Moges [25] in Bure district, Ethiopia. Suk and Park [26] reported positive correlations of YW, albumin weight (AW), and SW with EW. Similarly, Fanu et al. [21] noted positive correlations of EW with egg width, egg length, egg SW, and shell thickness. Bekele et al. [7] reported positive correlations of internal egg quality parameters.

## 5. Conclusions

The major poultry feeding practice in the study district was scavenging with additional supplementation. Maize and wheat are the main supplemented feeds. Majority of the respondents in the study area provide nighttime shelter to chickens. The egg production performance of exotic chicken breeds of SassoT44 was higher than that of KK in the study areas. Chicken producers in the area should be trained on improved chicken management practices which will be important to increase annual egg yields of the two chicken breeds to their genetic potential.

### 5.1. Future Research Directions

The future research directions include the following:

• Assessment and nutritional analysis of scavenging diets and identification of potential feed supplements is imperative in the future
• A comparative evaluation of the productivity of various native and exotic breeds under equivalent scavenging conditions is needed
• Additionally, assessment of the impact of housing systems to understand how shelter design and environmental conditions affect chicken health and egg production is required
• Offering trainings regarding the improved management practices focusing on feed formulation, disease management, and housing is crucial

### 5.2. Limitations of the Study

This study focuses exclusively on one district among the 22 districts in the South Wollo zone of Ethiopia. It specifically investigates two chicken breeds: SassoT44 and KK. Furthermore, the assessment of egg quality was performed with a limited sample size, constrained by financial limitations, which affected the overall extent of the research.

## Figures and Tables

**Table 1:** Household characteristics of respondents in the study area.

Variables		Production system		Total (*n* = 188)
		Urban (*n* = 80)	Rural (*n* = 108)	
Sex	Male	35 (43.8)	64 (59.3)	99 (52.7)
	Female	45 (56.3)	44 (40.7)	89 (47.3)

Marital status	Married	55 (68.8)	77 (71.3)	132 (64.9)
	Single	3 (3.8)	7 (6.5)	10 (5.3)
	Divorced	22 (27.5)	24 (22.5)	46 (18.6)

Educational status	Illiterates	10 (12.5)	19 (17.6)	29 (15.4)
	Read and write	31 (38.7)	34 (31.5)	65 (34.6)
	Elementary education	35 (43.8)	46 (42.6)	81 (43.1)
	High school	4 (5)	9 (8.3)	13 (6.9)

*Note: n* = number of respondents; the figures in parentheses are percentage.

**Table 2:** Number of chickens owned and keeping experience (frequency of households) in urban and rural production systems.

Variables		Production system		Overall
		Urban (*n* = 80)	Rural (*n* = 108)	
Chicken keeping experience (years)	1–3	52	76	128
	4–5	22	12	34
	> 5	6	20	26

Number of chicken breeds owned	5–10	71	96	167
	11–20	7	9	16
	> 20	2	3	5

**Table 3:** Chicken housing practices in urban and rural production systems of the study area.

Variables (%)	Production system		Total (*n* = 188)
	Urban (*n* = 80)	Rural (*n* = 108)	
*Available housing condition*			
Share the same house with family	11 (13.8)	34 (31.5)	45 (23.9)
Provision of separate night shelter only	61 (76.3)	54 (50)	115 (61.17)
Separate house constructed	6 (7.5)	3 (2.8)	9 (4.78)
Share the same house with other animals	2 (2.5)	17 (15.7)	19 (10.1)

*Reasons for not constructing a chicken house*			
Lack of knowledge	24 (30)	83 (76.9)	107 (56.91)
Lack of materials	24 (30)	18 (16.7)	42 (22.34)
Shortage of space	26 (32.5)	4 (3.7)	36 (15.95)

*Note: n* = number of respondents; the figures in parentheses are percentage.

**Table 4:** Feed source and feeding system in urban and rural production systems.

Variables (%)	Production system		Total (*n* = 188)
	Urban (*n* = 80)	Rural (*n* = 108)	
*Feeding system*			
Scavenging with supplement	63 (78.8)	86 (79.6)	149 (79.25)
Homemade feed	13 (16.3)	19 (17.6)	32 (17.02)
Purchased feed	4 (5)	3 (2.8)	7 (3.72)

*Supplement feed types*			
Maize and wheat grains	44 (55)	88 (81.5)	132 (70.2)
Wheat short	5 (6.3)		5 (2.66)
Kitchen waste	27 (33.8)	20 (18.5)	47 (25)

*Frequency of feeding*			
Morning and evening	5 (6.3)	21 (19.4)	26 (13.8)
Morning and afternoon	4 (5)	0	4 (2.66)
Morning, afternoon, and evening	71 (88.8)	87 (80.6)	158 (84.04)

*Note: n* = number of respondents; the figures in parentheses are percentage.

**Table 5:** Age at first lay, mature body weight, and egg production performances of SassoT44 and Koekoek chickens.

Parameters		Production system		Overall	*p* value
		Urban	Rural		
Age at first lay (month)	SassoT44	6.12^b^	6.70^a^	6.43	0.042
	Koekoek	6.00	6.40	6.18	0.41

Mature body weight (kg)	Sasso	2.71^a^	2.49^b^	2.59	0.043
	Koekoek	1.55	1.40	1.47	0.018

Total number of egg/year/hen	SassoT44	252.6^a^	226^b^	239.3	0.001
	Koekoek	198^a^	187.5^b^	193	0.07

^a,b^means within the same row with different superscript letters are significantly different (*p* < 0.05).

**Table 6:** Effects of chicken breeds and production system on external egg quality traits.

Variables		EW	EL	EWD	ESI	ESWT	EST
Chicken hybrids	SassoT44	56.7^a^	57.10	42.4^a^	72.00	6.20	0.29
	Koekoek	51.1^b^	55.11	41.5^b^	72.26	6.19	0.30
	SEM	0.68	0.51	0.25	0.71	0.15	0.015

Production area	Urban	56.16^a^	56.5	43.35^a^	74.00	6.21	0.37a
	Rural	50.12^b^	55.7	40.58^b^	70.10	6.10	0.22b
	SEM	0.68	0.54	0.23	0.50	0.12	0.025

*p* values	Breed (B)	0.001	0.23	0.038	0.71	0.65	0.81
	Production system (PS)	0.001	0.29	0.001	0.01	0.78	0.01
	B ∗ PA	0.004	0.21	0.14	0.73	0.5	0.56

Abbreviations: EL = egg length; ESI = egg shape index; EST = egg shell thickness, ESWT = egg shell weight; EW = egg weight; EWD = egg width.

^a,b^means along the same column with different superscripts are significantly different (*p* < 0.05).

**Table 7:** Effects of production system and chicken breeds on internal egg quality traits.

Variables		AW	AH	AR	YC	YW	YH	YR	HU
Chicken breeds	SassoT44	32.54^a^	5.88	0.57	7.29	18.53	18.53^a^	0.44^a^	82.01
	Koekoek	27.90^b^	5.50	0.56	8.56	18.40	17.45^b^	0.39^b^	81.1
	SEM	0.22	0.10	0.013	0.16	0.15	0.053	0.006	1.18

Production system	Urban	31.95^a^	5.84	0.56	7.23	19.84^a^	18.62^a^	0.42	80.12
	Rural	28.53^b^	5.53	0.55	8.62	17.09^b^	17.41^b^	0.41	81.9
	SEM	0.30	0.14	0.01	0.23	0.19	0.06	0.004	1.10

*p* values	Breed (B)	0.001	0.30	0.44	0.001	0.07	0.001	< 0.001	0.08
	Production system (PS)	0.001	0.67	0.49	< 0.001	0.001	0.001	0.43	0.10
	B ∗PS	0.001	0.01	0.09	< 0.001	0.01	0.377	0.089	0.32

Abbreviations: AH = albumen height; AR = albumen ratio; AW = albumen weight; YC = yolk color; YH = yolk height; YR = yolk index; YW = yolk weight.

^ab^means along the same column with different superscripts are significantly different (*p* < 0.05).

**Table 8:** Correlation coefficients among various egg quality traits.

Parameters	EW	EL	EWD	ESHI	ESW	EST	AW	AH	AR	YW	YH	YI	YR	YC	HU
Egg weight (EW)	1	0.59⁣^∗^	0.64⁣^∗^	0.62⁣^∗^	0.13⁣^∗^	0.52⁣^∗^	0.63⁣^∗∗^	0.17⁣^∗^	0.283	0.73⁣^∗∗^	0.76⁣^∗∗^	0.58⁣^∗^	0.57⁣^∗^	−0.43	0.73⁣^∗^
Egg length (EL)		1	0.67⁣^∗^	0.85⁣^∗∗∗^	0.16	0.7⁣^∗^	0.60⁣^∗∗^	−0.7	−0.16	0.40⁣^∗^	0.24⁣^∗^	0.56	−0.13⁣^∗^	0.20	0.43⁣^∗^
Egg width (EWD)			1	0.71	0.11	0.71⁣^∗^	0.72	−0.68	−0.43	0.52⁣^∗^	−0.56⁣^∗^	0.62⁣^∗^	0.40	0.37	0.61
Egg shape index (ESHI)				1	−0.05	0.93⁣^∗∗^	0.69⁣^∗∗^	−0.67⁣^∗^	−0.12	0.29	0.40	0.43	0.38	0.26	0.44⁣^∗^
Egg shell weight (ESW)					1	0.68⁣^∗^	0.017	0.42	−0.12	0.45	0.51⁣^∗^	0.49⁣^∗^	0.76⁣^∗∗^	0.51	0.56
Egg shell thickness (EST)						1	0.94⁣^∗∗^	0.55⁣^∗^	−0.37	0.38⁣^∗^	0.39	0.47	0.39	−0.41	0.16
Albumin weight (AW)							1	0.71⁣^∗∗^	−0.54	0.61⁣^∗∗^	0.15	0.44⁣^∗^	0.56⁣^∗^	−0.15	0.48
Albumin height (AH)								1	0.14	0.19	−0.47⁣^∗^	0.43	0.29	0.22	0.61⁣^∗^
Albumin ratio (AR)									1	−0.41	0.55	0.32	0.30	0.13	0.47
Yolk weight (YW)										1	0.63⁣^∗^	0.48⁣^∗^	0.65⁣^∗^	0.12	0.26
Yolk height (YH											1	0.69⁣^∗^	0.36	0.21	0.53
Yolk index (YI)												1	0.25	0.51	0.17
Yolk ratio (YR)													1	0.14	0.21
Yolk color (YC)														1	0.71
Haugh unit (HU)															1

⁣^∗^correlation is significant at *p* < 0.05 level.

⁣^∗∗^correlation is significant at *p* < 0.01 level.

⁣^∗∗∗^correlation is significant at *p* < 0.001 level.

## Data Availability

The data that support the findings of this study are available from the corresponding author upon reasonable request.

## References

[1] FAOSTAT (2020). *Food and Agricultural Organization of the United Nations*.

[2] Chebo C., Betsha S., Melesse A. (2022). Chicken Genetic Diversity, Improvement Strategies and Impacts on Egg Productivity in Ethiopia: A Review. *World’s Poultry Science Journal*.

[3] CSA (2021). Central Statistical Agency: Agricultural Sample Survey Volume II Report on Livestock and Livestock Characteristics. *Addis Ababa, Ethiopia*.

[4] Entity M. (2021). Livestock Systems: Overview and Areas of Inquiry. *Gainesville, FL: Feed the Future Innovation Lab for Livestock Systems*.

[5] Urgesa L. (2023). Review on Poultry Production System, Trends and Development Strategies in Ethiopia. *Journal of Aquaculture & Livestock Production*.

[6] ENTAG (2020). Invest in the Ethiopian Poultry Sector. *Business Opportunity Report*.

[7] Bekele B., Melesse A., Esatu W., Dessie T. (2021). Production Performance and Egg Quality Evaluation of Indigenous Chickens Across Different Agro-Ecologies of Southern Ethiopia. *Veterinary Integrative Sciences*.

[8] Erdaw M. M., Beyene W. T. (2022). Trends, Prospects and the Socio-Economic Contribution of Poultry Production in Sub-Saharan Africa: A Review. *World’s Poultry Science Journal*.

[9] JDAO-Jama district administration office (2019). Jama District Administration Office, Annual Summery Report.

[10] Yamane T. (1967). *Statistics: An Introductory Analysis*.

[11] Ebsa Y. A., Harpal S., Negia G. G. (2019). Challenges and Chicken Production Status of Poultry Producers in Bishoftu, Ethiopia. *Poultry Science*.

[12] Matawork M., Meseret M., Samuel T. (2019). Productive and Reproductive Performance of Indigenous Chickens in Gena Bossa District of Dawro Zone, Ethiopia. *International Journal of Livestock Production*.

[13] Habtie A. (2019). *Characterization of Chicken Production System and On-Farm Evaluation of Introduced Exotic Chicken Breeds in Gondar Zuria and Kalu Districts of Amhara Region*.

[14] Nega M., Fekadu B., Senayt A. (2017). Husbandry Practices and Egg Production Performances of Exotic Chicken Breeds in Assosa Town, Beneshangul Gumuze Region, Ethiopia. *Advance Research Journal of Multidisciplinary Discoveries*.

[15] Ayalew H., Chanie D., Fentahun T., Yinnesu A., Dagnew Y., Moges D. (2023). Agro-Ecological Base Differences of Village Based Local Chicken Performance and Household Product Consumption in Amhara Region, Ethiopia. *Cogent Food and Agriculture*.

[16] Suntebo M. M. (2023). Assessment of Chicken Husbandry Practices in Selected Districts of Gamo Zone, Southern Nation Nationality and Peoples Region, Ethiopia. *International Journal of Science and Research Archive*.

[17] Mohammed A. (2018). Major Constraints and Health Management of Village Poultry Production in Ethiopia: Review School of Veterinary Medicine, Jimma University, Jimma, Ethiopia. *Journal of Research Studies in Microbiology and Biotechnology*.

[18] Aman G., Addisu J., Mebratu A., Kebede H., Bereket Z., Tekla B. (2017). Management Practices and Productive Performances of Sasso Chickens Breed Under Village Production System in SNNPR, Ethiopia. *Journal of Biology, Agriculture and Healthcare*.

[19] Gonta E., Urge M., Girma M. (2022). Assessment of Smallholder Chicken Feeding Practices, Available Feeds, and Evaluation of Locally Used Mixtures in Two Districts of South Omo Zone, Ethiopia. *Veterinary Research Notes*.

[20] Tadesse D., Singh H., Esatu A. M. W., Dessie T. (2013). Study on Productive Performances and Egg Quality Traits of Exotic Chickens Under Village Production System in East Shewa, Ethiopia. *African Journal of Agricultural Research*.

[21] Fanu W. M., Melkamu B. Y., Emana G. (2019). Productivity and Egg Quality Traits of Sasso T44 Chicken in North Showa Zone, Ethiopia. *Journal of Animal and Plant Sciences*.

[22] Tadesse D., Esatu W., Girma M., Dessie T. (2015). Comparative Study on Some Egg Quality Traits of Exotic Chickens in Different Production Systems in East Shewa, Ethiopia. *African Journal of Agricultural Research*.

[23] Kumar N., Belay Z. N., Asfaw Y. T., Kebede E. (2014). Evaluation of Egg Quality Traits of Rhode Island Red and Bovans White Under Intensive Management in Mekelle Ethiopia. *IOSR Journal of Agriculture and Veterinary Science*.

[24] Nebiyu Y. A. (2016). Assessment of Urban Poultry Production Practices in Addis Ababa With Emphasis on Egg Production, Product Marketing, Feed Quality and Waste Management. *PhD Dissertation Department of Animal Production Studies, College of Veterinary Medicine and Agriculture*.

[25] Moges F. (2010). Indigenous Chicken Production and Marketing Systems in Ethiopia: Characteristics and Opportunities for Market-Oriented Development.

[26] Suk Y. O., Park C. (2001). Effect of Breed and Age of Hens on the Yolk to Albumen Ratio in Two Different Genetic Stocks. *Poultry Science*.

